# Using of non-pharmacological pain methods, and the perceived barriers, among nurses in critical care unit in Palestine

**DOI:** 10.1186/s12912-023-01635-9

**Published:** 2023-12-07

**Authors:** Wafaa Jameel Tohol, Ashraf Jehad Abuejheisheh, Imad Fashafsheh, Nawaf Amro

**Affiliations:** 1 Arab American University, Ramallah, Palestine; 2 Al-Quds University, Jerusalem, Palestine; 3 Modern University College, Ramallah, Palestine

**Keywords:** Non-pharmacological pain methods, ICU, Nurses, Barriers to implement non-pharmacological pain methods, Nurse’s practice of non-pharmacological pain methods

## Abstract

**Background:**

Pain is alleviated in one of the two common approaches, pharmacological or non-pharmacological intervention. Using non-pharmacological methods may have beneficial effect and priority on decreasing patients’ level of pain and decreasing the side effects of pharmacological methods in ICU patients. This study aimed to assess ICU Palestinian nurses use and barriers of non-pharmacological pain management.

**Methods:**

A quantitative descriptive cross-sectional design was used to collect responses from a convenient sample of 215 nurses working in six hospitals in Plaestine. The timeframe for data collection was from August 2022 to December 2022. This study had three instrument packages. The first part is demographic data. The second is a tool that used 4-point Likert scale to examine ICU nursing use of non-pharmacological pain methods and it consisted of 16 items. The third is six items of perceived barriers to use non-pharmacological pain methods. All statistical procedures were analyzed using Statistical Package of Social Science (SPSS) version 28.

**Results:**

More than two-thirds of the nurses used non-pharmacological pain methods in ICU. 60% of the nurses have high levels of use, 10.2% have very high levels. The two highest perceived barriers to use nonpharmacological method were the lack of time, workload, and patient instability with 83.7% (n = 180), and 77.2% (n = 166), respectively. Demographic variables were not significantly associated with the use of non-pharmacological pain management methods, except age.

**Conclusion:**

Adopting culturally sensitive non-pharmacological pain methods to decrease ICU patients’ level of pain, may positively reflected on patients’ outcome and on healthcare system. Developing, implementing and continuous monitoring of guidelines regarding using nonpharmacological for nurses and physicians are recommended which will be reflected positively on patients’ outcomes. Great efforts to overcome the barriers of lack of time and workload is impertive to increase the clinical usage of nonpharmacological pain methods.

## Introduction

Millions of people worldwide are suffering from pain, whether they are in the hospital, their homes or assisted living facilities [1]. The opioid crisis is a nationwide emergency that is leading to addiction, overdose, and death. Nonpharmacological pain treatment approaches have a lot of research backing them up, yet nurses rarely use them in clinical practice [2]. It is imperative for ICU nurses to have an in-depth understanding of the non-pharmacological pain methods. Utilizing the non-pharmacological pain methods by ICU nurses may decrease the side effects of the pharmacological methods which may improve patient outcomes and increase quality of life [3].

Pain is defined by The International Association for the Study of Pain as “an unpleasant sensory and emotional experience associated with, or resembling that associated with, actual or potential tissue damage.” [4]. Pain is also define as whatever the patient says it is, and it exists whenever the patient says it does [5]. Pain in the critically ill is linked to negative physiological and psychological outcomes [6] and it has an impact on the quality of life of patients when they are discharged from the intensive care unit (ICU) [7, 8]. Despite significant progress in critical illness pain evaluation and analgesia, pain in critical illness remains an unsolved topic [9, 10].

There are many studies worldwide and nationally highlighted the high number of patients expereicning pain who need strategies to decrease its level. For instance, Presently, approximately 80% of the world’s population is believed to experience insufficient pain management, presenting a significant challenge in over 150 nations [11]. Futhermore, a systematic review that included randomized controlled trials (RCTs) and observational investigations conducted with the primary objective of identifying the incidence or prevalence of persistent post-ICU pain revlealed that the incidence or prevalence ranged from 28 to 77% at a minimum of three months following discharge from the intensive care unit (ICU) [12]. However, in Palestine, and up to the researchers’ search no studies or statistics on the pain in ICU were found. On the othert hand, The findings of a study conducted in Palestine by Salameh [13] indicated that nurses with high acuity levels demonstrated insufficient understanding in both pharmacological and non-pharmacological approaches to pain management, along with a lack of knowledge in addressing patient pain. This knowledge defict is supported by another study that revealed the overall mean score of the level of knowledge about pain management among nurses was 15.5 out of 34 (45.6% out of 100%) [14]. Knowledge was the strongest predictor for ICU nurses to practice or use of evidence based research such as pain amangement [15].

Pain is alleviated in one of the two common approaches, pharmacological or non-pharmacological intervention. The first method is widely used to decrease the level of client’s pain by administering the wide-range regimens of opioids such as morphine, fentanyl or non-opioids such as Nonsteroidal Anti-Inflammatory Drugs (NSAID) [16]. However, and even though these drugs are necessary for relieving pain in ICU patients, they have side effects such a higher risk of delirium, hypotension, and respiratory failure [17]. Therefore, looking for other less-side effect interventions to alleviate pain is crucial such as non-pharmacological pain management.

Non-pharmacological pain management interventions divided into three main categories [18]. The first one is physical interventions, like massage, positioning, heat and cold therapy, transcutaneous electrical nerve stimulation (TENS), acupuncture, and progressive muscle relaxation. The second one is psychological therapy. The third type is other therapies, including spirituality and religious activities as well as music therapy and listening to Qur’an [18, 19].

Using non-pharmacological methods, may have beneficial effect on patients’ level of pain by reducing it and decreasing the chance of side effects arising from pharmacological method [3]. Moreover, when non-pharmacological methods are utilized, the pharmacological intervention may be reduced or even substituted by the non-pharmacological methods which may decrease harm on patients and increase quality of life [20, 21]. Pain should be treated with a multimodal approach that includes both pharmacologic and nonpharmacologic treatments [22]. In addition, non-pharmacological interventions also yield other benefits, such as lower medical costs, greater availability to patients, diversification and ease of use, and greater patient satisfaction [3].

There are many barriers to implementing non-pharmacological methods to decrease pain. Lack of education and high nurse workload are two examples of barriers. First, lack of education is one of which has been shown in literature that a lack of information among both health care personnel and patients is one of the most significant hurdles to treating pain in ways other than medications. Plaisance and Logan [23] found that, despite major efforts from statewide Pain Initiatives and certifying organizations, knowledge of pain treatment is still inadequate, and that further education is needed. Patients’ strong belief in only pain medication, according to Ambola, Ajong [24], was the number one reason for not requesting or trusting nonpharmacologic methods of pain management when polled through a questionnaire.

Pain management education for nurses and nursing students is also needed. Stewart and Cox-Davenport [25], nurse researchers, investigated how nurses and nursing students feel about applying nonpharmacologic pain therapies. Only 65% of nursing students and 51% of nurses said they were sufficiently educated on the subject. The fact that just half of registered nurses believe they are informed on this topic, so it is important to provide a comprehensive patient care and highlight the need for further education.

Regarding high nurse workloads, a literature review completed by Gumus, Musuroglu [26] pointed out that in general, the factors that complicate and prevent the use of the nonpharmacologic methods are not merely limited to a lack of relevant education. Although that is the largest issue overall, other barriers include high nurse workload, desire to control acute pain as quickly as possible, and not having the available resources. 40% of nurses in a descriptive study with self-administered questionnaire stated that their workload is too high to regularly implement nonpharmacologic methods of pain management [24]. Another study conducted by Khalil [1] has found the same findings as Ambola, Ajong [24] that the lack of time was the most significant obstacle to implement non-pharmacological interventions. In Palestine, and up to the researcher knowledge, no studies were found to examine using nonpharmacological pain management.

ICU nurses play a pivotal role in the holistic care of patients, but there is a gap in the consistent implementation and integration of nonpharmacological pain management techniques into their daily practices [27]. This gap poses several issues, such as inadequate pain relief, prolonged recovery times, increased risk of complications, and decreased overall patient satisfaction [28]. Additionally, the overreliance on pharmacological interventions can lead to adverse effects, drug interactions, and potential dependency issues. Therefore, it is imperative to address this gap and explore the barriers preventing ICU nurses from utilizing nonpharmacological pain management methods to their full potential.

Understanding the reasons behind the underutilization of nonpharmacological pain management techniques among ICU nurses is essential for enhancing patient care and outcomes [29]. By identifying the uses of nonpharmacological pain methods, the following important purposes will consider such as enhancing patient comfort and experience, minimizing pharmacological interventions which consequently reduced complications, provide a holistic patient care and reducing healthcare costs [30].

One of the impetuses to conduct this research was the clinical experience of the researchers in Palestine, where scarce nonpharmacological methods to decrease pain were noticed to be used by nurses in ICU and other departments. Therefore, the aims of this study were to assess ICU Palestinian nurses use of non-pharmacological pain management modality to decrease ICU patients’ pain, and to examine the barriers to implementing this modality.

## Method

### Research design

A descriptive design using cross-sectional survey was used for the purpose of this study. A survey was used at point of time to examine subjects’ use of non-pharmacological pain management and barriers among ICU nurses to decrease ICU patients’ pain level. Descriptive design is suitable for this study because the purpose is to describe and document aspects of using non-pharmacological pain management and barriers, and it is efficient in collecting large amount of data in short time about the problem [31]. Descriptive design does not focus on examining causal relationship as experimental designs which is not among the aims of this study.

### Setting

According to the Ministry of Health (MOH) in Palestine, hospitals are divided into four main sectors: Governmental, private, non-governmental organizations (NGOs), and educational hospitals [32]. The researcher selected six major hospitals from West Bank-Palestine. One large private hospital and one large governmental hospital from each of the north, middle and south of West Bank-Palestine. The reason behid using these hospital is that they are the largest in West Bank and the existence of large number of ICU beds. These hospitals are X1 Governmental Hospital {20 ICU beds} and X2 Private Hospital {14 ICU beds} from the city of Nablus in the north; Y1 Governemntal {44 ICU beds} and Y2 Private Hospital {12 ICU beds} from the city of Ramallah in the Central West Bank as well as the Z1 Governmental Hospital {16 ICU beds} and Z2 Private hospital {26 ICU bes} from the city of Hebron in the south. All ethical approvals were obtained before starting data collection.

### Sampling

The target population for this study has included all Palestinian nurses working in ICUs while the accessible population has included nurses working in the ICUs in the six selected hospitals. The inclusion criteria for nurses were as follows; holding a Diploma in nursing, having a minimum of one-year experience in ICUs, full-time employees as practicing nurses. Exclusion criteria included administrators. Nurses were conveniently recruited. Although the Ministry of Health (MOH) in Palestine was contacted and searched via its official website, no data on the accurate number of all ICU nurses in Palestine were available to calculate the sample size. For this reason, the researchers contacted all hospitals and visited some of them for the purpose of calculating the number of ICU nurses in each hospital. The researchers met one of the administrator hospitals and revealed that the entire population was 500 ICU nurses.

### Sample size

**Fig. 1:**
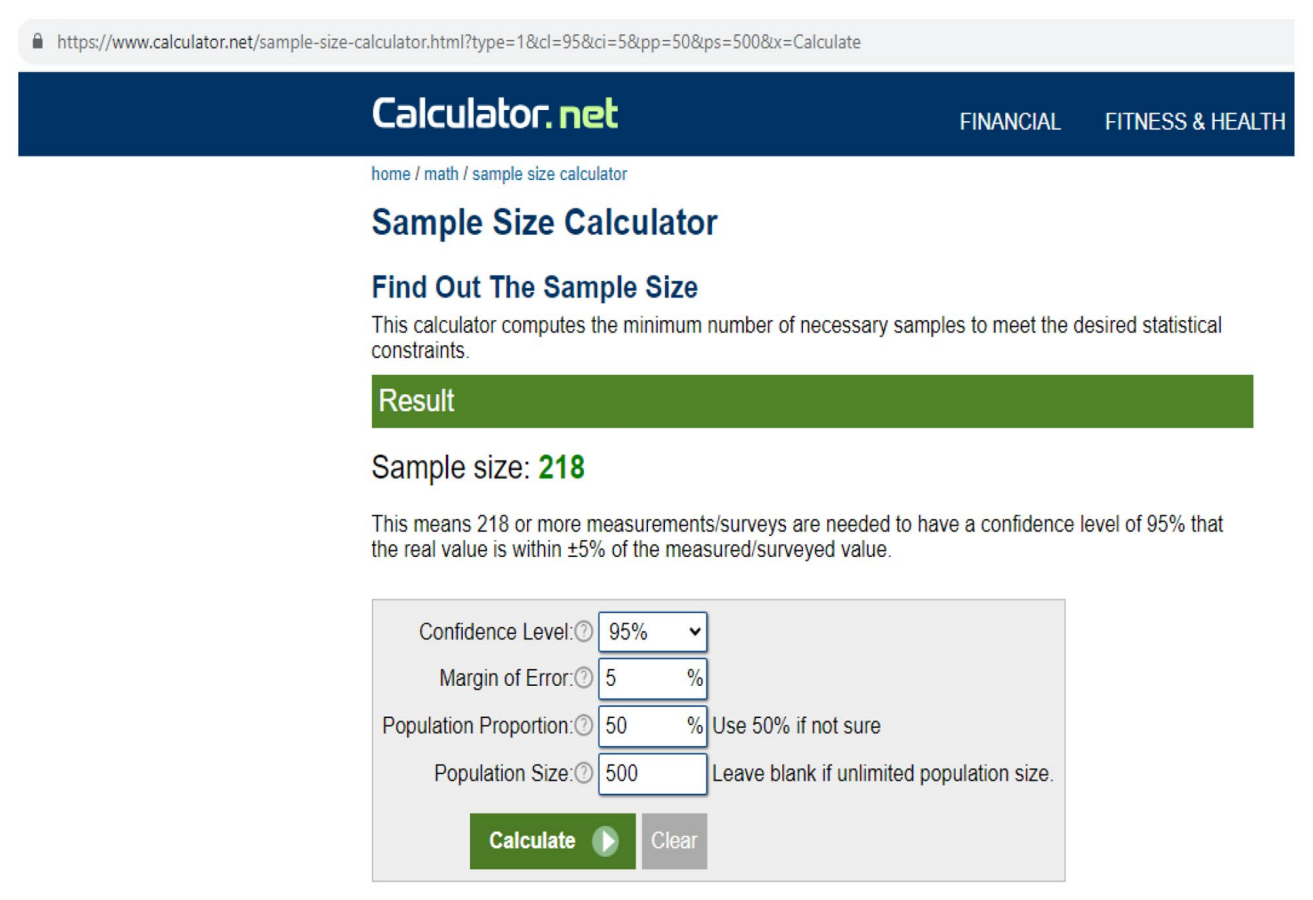
Calculation of sample size using online sample size calculator. Confidence interval 95%, margin of error 5% and population of 500 ICU nurses. The required sample size was 218. Accessed from https://www.calculator.net/sample-size-calculator.html?type=1&cl=95&ci=5&pp=50&ps=500&x=Calculate

The sample size was calculated according to the online sample size calculator formula with consideration to the confidence interval 95%, margin of error 5% and population of 500 nurses. The required sample size was 218, we added 32 particiapnts to overcome possibility of incomplete questionnaires and attrition. However, 215 nurses were completed the questionnaire, giving a response rate of 86% [33]. A screeshot for the utilized formula for sample size calculation is attached below in Fig. 1.

### Instrument

This study had three instrument packages. The demographic data, the use of nonpharmacological pain method, and the barriers to use nonpharmacological pain method. The first section established by the researcher includes demographic variables such as sex, age, level of education, years of experience as a nurse, years of experience as ICU nurse, working area (type of ICU), health work sector, education source on non-pharmacological pain methods.

The second section was a tool that used a 4-point Likert scale to examine ICU nursing use of non-pharmacological pain methods. This tool was developed and created by Khalil [1] after a literature review. It consisted of a list of 16 intervention methods related to non-pharmacological pain methods). This tool consisted of 16 items, each of which required responses to be recorded on a four-point Likert scale, which ranged from 1 (never), 2 (few times), 3 (sometimes), and 4 (frequently). In term of scoring system, the 4-point Likert scale divided into four ranges, and to illustrate the mean scores cut-off they categorized into these four levels (Low mean score = 1-1.75, moderate = 1.76–2.51, high = 2.52–3.27, and very high = 3.28-4). The tool was reviewed and validated by a panel of experts in critical care nursing and pain management. Test and retest were carried out, and the correlation coefficient was 0.7 [1]. Furthermore, the intra class correlation coefficient of this tool was 0.99 by Iranian researcher who used the same tool, but they translated it to Persian language [3]. In addition, the researcher calculated the reliability (reliability coefficient) of the non-pharmacological pain methods items for the sample of this study and the Cronbach’s Alpha of the items was 0.84 for the participants of this study, which gives an acceptable internal consistency. Table 1 shows the reliability coefficient of the non-pharmacological pain methods items. This reliability was calculated via SPSS version 28.0. Items for using nonpharmacological pain management were inserted to the SPSS and the results are shown in Table 1 below.

**Table 1:** *Reliability coefficient*

Variables	n	Cronbach’s Alpha
Non-pharmacological pain methods items	16	0.835

The third and the last section of the instrument package was the barrier tool. In this section, six items of perceived barriers to use non-pharmacological pain methods were detected and nurses were asked to state whether these items are barriers to use these methods, or they are not barriers [1].

### Ethical considerations

Approval forms the Scientific Research Committee at the School of Nursing-The Arab American University Palestine was obtained. In addition, the approvals from the Ethical Committees at each selected hospital were obtained before data collection. The subjects’ permission was received after meeting and before recruiting them. The researcher has explained to nurses the purpose of the study and the subjects’ rights were preserved. They were informed that participation in this study is voluntary, and the researcher will maintain the anonymity by recording no personal identification. Moreover, detailed information about the objectives of the study, the needed time to complete the questionnaire were contained in a cover letter (the maximum time needed is 15 min), which was attached at the beginning of the questionnaire. The data collection took place in the ICUs of the selected hospitals, and the questionnaires were collected from each participant by the researchers. The researchers expressed their thanks and appreciation for each participant for taking part in this study. Only the researchers had access to the questionnaire. (Code for all Governmental hospitals: 162/1811/2022). For the private hospitals the form of approval exists and will be provided upon request.

### Data collection procedure

When the required ethical approvals were obtained, the researchers made an appointment with nursing directors of each selected hospital and met them to introduce themselves to the, explain the purpose of the current study, and to facilitate the approach to head nurses and nurses in ICUs. After that, the researchers met all nurses (as a group) who met the eligibility criteria in a special room. The purpose of the study was explained, and they were invited to participate in the study. Verbal permission was obtained from each subject who wants to participate in this study. Then, questionnaires were distributed to the eligible subjects who verbally confirmed their participation in this study. The researchers were available in ICUs next to nurses for any clarification, explanation, and questions from nurses regarding the questionnaires and the study. Then the questionnaires were distributed to the targeted sample. It took each nurse about 8 min to complete the questionnaire. When the subjects finished filling out the questionnaires, the researchers collected them, and they expressed thanks and appreciation for their participation and efforts. The timeframe for data collection was from August 2022 to December 2022.

### Data analysis

All statistical procedures were analyzed using Statistical Package of Social Science (SPSS) version 28 [34]. The assumptions for each test were checked before carrying out the test. Descriptive statistics were conducted to calculate means, Standard Deviation (SD) and frequencies of the study variables. Furthermore, Independent t-test was conducted to compare using nonpharmacological pain methods among male and female while one way analysis of variance (ANOVA) was used to compare the level of using nonpharmacological pain methods among the variables that have three or more options (i.e., level of education) [35]. Data was tested for normality using the Shapiro-Wilk tests. Table 2 presents the Shapiro-Wilk tests which shown that the total mean score was normally distributed (*p* = 0.171). The significance level was set to 0.05.

**Table 2:** *Tests of Normality*

Variable	Shapiro-Wilk test		
	Statistic	df	Sig.
Total mean score of using non-pharmacological pain methods	0.990	215	0.171

## Results

Table 3 presents the socio-demographic characteristics of the nurses in the ICU. Out of 250 questionnaires, 215 were obtained giving a response rate of 86%. 60.9% of the nurses were males while the rest were females. More than two-thirds of the nurses hold a bachelor’s degree. In addition, more than half of the nurses have 1–5 years of professional experience in nursing. Most of education sources on non-pharmacological pain management were from books and colleagues with 37.7% and 32.6% respectively. More details are shown in Table 3.

**Table 3:** *Socio-demographic characteristics of the nurses in the ICU (n = 215)*

Variables		n	%
Gender	Male	131	54.6
	Female	84	39.1
Age groups	20–25 years old	86	40.0
	26–30 years old	58	27.0
	31–35 years old	47	21.9
	36–40 years old	13	6.0
	41–45 years old	11	5.1
Level of education	Diploma	30	14.0
	Bachelor’s Degree	167	77.7
	Master’s degree	18	8.4
Years of experience in nursing	1–5 years	121	56.3
	6–10 years	50	23.3
	11–15 years	26	12.1
	16–20 years	10	4.7
	21–25 years	8	3.7
Years of experience in ICU	1–5 years	162	75.4
	6–10 years	34	15.8
	> 10 years	19	8.8
Working area (type of ICU)	Surgical ICU	65	30.2
	Medical ICU	81	37.7
	CCU	69	32.1
Health sector type	Governmental	114	53.0
	Private	101	47.0
Which education source on non-pharmacological pain management you have	Opinion	22	10.2
	Book	81	37.7
	Colleague	70	32.6
	Published work (articles)	15	7.0
	In-service education	20	9.3
	None	7	3.3

**Fig. 2:**
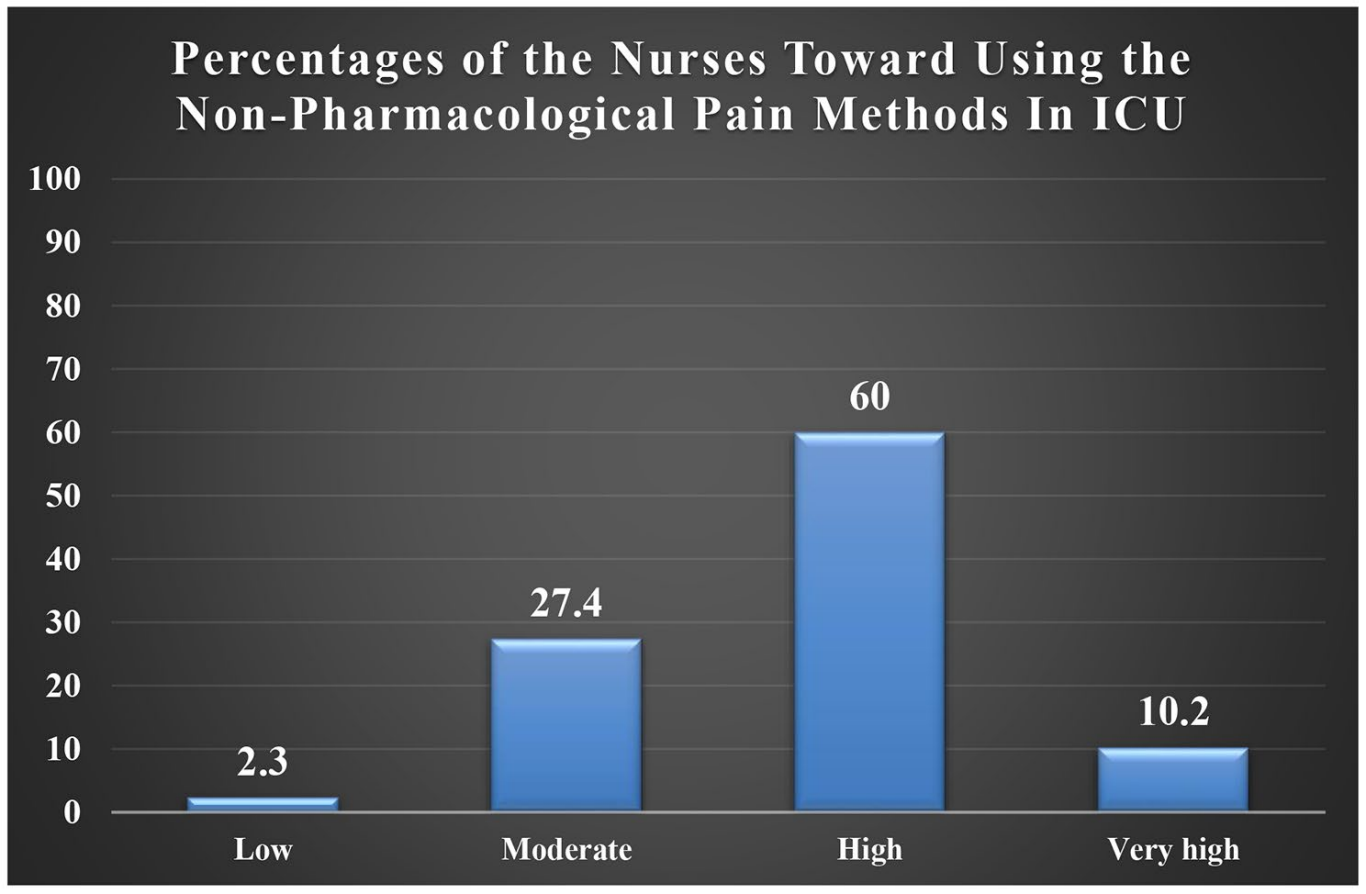
Percentages of Nurses that have used Non-Pharmacological Pain Methods in ICU

Figure 2 illustrates the percentages of nurses that have used non-pharmacological pain methods in ICU. More than two-thirds of the nurses used non-Pharmacological Pain Methods in ICU. 60% of the nurses have high level, 10.2% have very high level. On the other hand, 27.4% are moderate and 2.3% are low.

Table 4 shows the mean score for each item relevant to non-pharmacological pain management methods. The total mean score for all times was 2.71 (SD, 0.46). The highest methods used by nurses in terms of non-pharmacological pain management were placing the patient in a comfortable position and communicating with the patient & family with M = 3.40, SD = 0.801 and M = 3.27, SD = 0.764 respectively.

**Table 4:** Mean score for each item toward non-pharmacological pain management methods (n = 215)

Item	M	SD	%
Put the patient in comfortable position	3.40	0.801	85.0%
Apply hot or cold local packages	2.79	0.778	69.8%
Encourage patient to drink herbal drinks	2.62	0.904	65.5%
Apply breathing techniques	3.08	0.864	77.0%
Conduct hydrotherapy (partial bath)	2.77	0.948	69.3%
Apply movement restriction-resting	2.81	0.795	70.3%
Communicate with patient, &family	3.27	0.764	81.8%
Use therapeutic touch	2.63	0.897	65.8%
Apply massaging techniques	2.51	0.814	62.8%
Distract the patient by listening to light music/watching TV	1.97	1.016	49.3%
Help the patient to pray	2.64	0.885	66.0%
Apply guided imagery technique	2.27	0.904	56.8%
Provide quiet and comfortable room	2.92	0.893	73.0%
Use comfort devices (special mattress)	2.85	0.920	71.3%
Counselling/Provides education for patient and his family	3.00	0.788	75.0%
Acupuncture/acupressure/reflexology	1.98	0.888	49.5%
Total Mean Score (16 items)	2.71	0.465	67.8%

However, the lowest methods used were Distracting the patient by listening to light music/watching TV and acupuncture/acupressure/reflexology with M = 1.97, SD = 1.016 and M = 1.98, SD = 0.888 respectively. More details are shown in the Table 4.

Table 5 presents the barriers to use nonpharmacological pain methods among ICU nurses to decrease ICU patient level of pain. As shown in Table 5, the two highest barriers were the lack of time, workload and patient instability with 83.7% (n = 180), and 77.2% (n = 166), respectively. On the other hand, lack of education and low priority of pain management were considered as the two lowest barriers according to the participants’ responses with 49.3% (106), and 54.4% (117), respectively.

**Table 5:** Barriers to use non-pharmacological pain management among ICU nurses (n = 215)

Item	Yes		No	
	n	%	n	%
Lack of time and high workload	180	83.7	35	16.3
Lack of equipment	153	71.2	62	28.8
Lack of education	106	49.3	109	50.7
Patient instability	166	77.2	49	22.8
Patient inability to communicate	148	68.8	67	31.2
Low priority of pain management	117	54.4	98	45.6

Table 6 shows the differences among socio-demographic characteristics in terms of the total mean score of using the non-pharmacological pain management among ICU nurses. The Independent t-test and One Way ANOVA were used to assess the differences among variables. The Independent t test has shown that there are no significant differences in gender (*p* = 0.090) and types of hospitals (*p* = 0.574). In addition, One Way ANOVA has shown that there are no significant differences in level of education (*p* = 0.292), years of experience in nursing (*p* = 0.252), years of experience in ICU (*p* = 0.964), work area (*p* = 0.666) and education source (*p* = 0.627).

**Table 6:** Differences among socio-demographic characteristics in terms of the total mean score of using the non-pharmacological pain management among ICU nurses (n = 215)

Variables		n	Mean	SD	Statistical value	*P*-value
Gender	Male	131	2.67	0.457	T = -1.703 df = 213	0.090
	Female	84	2.78	0.473		
Age groups	20–25 years old	86	2.83	0.456	F = 2.633 df = 4	0.035*
	26–30 years old	58	2.59	0.403		
	31–35 years old	47	2.68	0.454		
	36–40 years old	13	2.65	0.573		
	41–45-year-old	11	2.64	0.620		
Level of education	Diploma	30	2.84	0.544	F = 1.238 df = 2	0.292
	Bachelor’s Degree	167	2.70	0.453		
	Master’s degree	18	2.67	0.428		
Years of experience in nursing	1–5 years	121	2.76	0.436	F = 1.351 df = 4	0.252
	6–10 years	50	2.66	0.470		
	11–15 years	26	2.71	0.477		
	16–20 years	10	2.46	0.494		
	21–25 years	8	2.62	0.726		
Years of experience in ICU	1–5 years	162	2.71	0.468	F = 0.037 df = 2	0.964
	6–10 years	34	2.71	0.385		
	> 10 years	19	2.74	0.584		
Working area (type of ICU)	Surgical ICU	65	2.75	0.506	F = 0.407 df = 2	0.666
	Medical ICU	81	2.68	0.366		
	CCU	69	2.73	0.529		
Working health sector	Government	114	2.70	0.500	T = − 0.564 df = 213	0.574
	Private	101	2.73	0.425		
Which education source on non-pharmacological pain management you have	Opinion	22	2.72	0.372	F = 0.697 df = 2	0.627
	Book	81	2.70	0.459		
	Colleague	70	2.76	0.462		
	Published work (articles)	15	2.82	0.400		
	In-service education	20	2.62	0.586		
	None	7	2.52	0.626		

*Significant at *p* = ≤ 0.05

Independent t test and one way ANOVA

However, a significant difference between age groups was found (F = 2.633, *p* = 0.035). According to the Tukey post-hoc test, ICU nurses who aged between 20 and 25 years old (M = 2.83, SD = 0.456) have higher mean score than nurses aged between 26 and 30 years old (M = 2.59, SD = 0.403) (*p* = 0.020).

## Discussion

The results of this study showed the percentages of nurses using the non-pharmacological pain methods in ICUs. More than two-thirds of nurses were using the non-Pharmacological Pain Methods in ICU. 60% of nurses have used these methods at a high level, 10.2% at a very high level while 27.4% at a moderate level and 2.3% at a low level. In the same context, Kia, Allahbakhshian [3] as shown in our result revealed that a moderate number of ICU nurses used non-pharmacological pain management methods (55.8%) out of 224 ICU nurses. In the same context, the more cognizant nurses are of spirituality and spiritual care as one of the nonpharmacological methods to decrease pain, the more effective care and interventions they can deliver to their patients. A study conducted in Iran by Abdollahyar et al. [19] pointed out that the attitude of the nurses regarding spirituality and spiritual care was in a relatively favorable spectrum with 84.8% of the 125 partipating nurses. Emphasizing the significance of maintaining a positive attitude when addressing patients’ pain is crucial, as it serves as a pivotal factor and a robust predictor for the successful implementation of pain management practices, encompassing both pharmacological and nonpharmacological methods [15].

On the other hand, Khalil [1] and Zeleke, Kassaw [5] reflected inconsistent results in our study as they concluded that most nurses didn’t apply non-pharmacological pain management approaches. For instance, only 26% nurses used nonpharmacological pain methods. An interesting point in our study and the two previously mentioned studies is that the ICU nurses used placing the patients in a comfortable position as the most frequent method to decrease their pain. Some difference in the location of these two studies and our study may influence the results. For example, our study was conducted in a variety of health sector hospitals in Palestine, while Khalil [1] study was conducted in a single hospital in Egypt, and Zeleke, Kassaw [5] study was conducted in a single hospital in Ethiopia. Moreover, the sample size included 60 nurses and 169 nurses in Khalil [1] and Zeleke, Kassaw [5] respectively, while our sample size was larger, and it included 215 ICU nurses.

Khalil [1] stated that the lack of education and inadequate knowledge were a frequent barrier that prevented critical care nurses from applying non-pharmacological pain management approaches. This was incongruent with the participants of our study who claimed that lack of education was the lowest barrier to use nonpharmacological pain methods to decrease ICU patient pain. This may give the impression that nursing education in Palestine have emphasized using nonpharmacological pain methods rather than the case in Egypt and Ethiopia. According to The World Bank classification, Palestine is a lower-middle income economy while Ethiopia is a low-income economy, which may be considered as another reason [36]. Effective non-pharmacological treatment must be implemented by trained, competent nurses. Numerous research has revealed that in order to lessen patients’ pain intensity, nurses need to be better knowledgeable about non-pharmacological pain treatment [37, 38]. Thus, it is imperative that good knowledge is a staring to enhance nursing practice of non-pharmacological pain treatment.

The lack of time and nurses’ high workload were the most perceived barrier to use nonpharmacological pain method by ICU nurses to decrease ICU patient level of pain in the Iranian study [3]. These results go along with our study result which revealed that approximately 84% of participants have the same barrier.

Regarding the demographics, our study pointed out no statistically significant relationships between using the nonpharmacological pain method and sex, years of experience, working area and level of education. Our findings are consistent with [1, 3]. In term of age, Khalil [1] revealed that a few nurses with more experience (over 20 years) used more nonpharmacological pain intervention practices than those with 2–4 years of experience. On contrast, the present study stated that the age group between 20 and 25 years have higher mean score to use nonpharmacological pain method.

### Implications and recommendations

The findings of this study could be beneficial to the clinical area where they could be applied and may give some recommendations regarding this vital concern. The results regarding using a nonpharmacological method and the barriers raised can be used as a base line data for healthcare professions, and more attention can be paid for using a more structural guidelines regarding this concern. Other future studies with different designs, such as observational and interventional studies, are needed to explore this issue, and the hospital staff in Palestine are recommended to use these studies and they are recommended to have a large sample size to increase the representativeness and generalizability of results. Also, nurses’ practice regarding using nonpharmacological care should be studied with other designs to assure that nurses implement such an approach as well as monitor the patients’ outcome. Finally, we strongly recommend involving ICU patients in future studies to examine the efficacy of different nonpharmacological pain methods on their level of pain in robust designs such as randomized controlled trials.

### Limitations and strengths

Few limitations may have effect on the findings of this study. Although the researchers strive to find the exact number of ICU nurses in Palestine, no information was found by official institutions such as MOH in Palestine. However, the researchers visited and made phone calls with the administrators of each hospital and obtained the exact number of each ICU nurses in Palestine. The convenience sampling technique was used to approach ICU nurses, which may have a possibility of bias. Also, the data were collected form nurses to assess their usage of nonpharmacological pain method through the self-administered questionnaire, but this self-reporting method may have a possibility of bias. On the other hand, up to the researcher knowledge, this is the first study that have been conducted in Palestine which is a strength and an added value to our study. All studies that have been found concerning this issue were conducted in a single hospital and they focused on general nurses or al the nurses in the hospital, but our study has focused on ICU nurses rather than general nurses or the registered nurses in the hospital entirely, which is also another strength to our study.

## Conclusion

This study is the first one in Palestine that assessed using nonpharmacological pain management and addressed the barriers among ICU nurses. Adopting culturally sensitive nonpharmacological pain methods to decrease ICU patients’ level of pain, may positively reflected patients’ outcome on healthcare system. Developing, implementing and continuous monitoring of guidelines regarding using nonpharmacological for nurses and physicians are recommended which will be reflected positively on patients’ outcomes. It is strongly recommended to involve ICU patients in future interventional studies to examine the efficacy of different nonpharmacological pain methods.

## Data Availability

The datasets used or analyzed during the current study are available from the corresponding author upon reasonable request. The data are not publicly available due to privacy and ethical restrictions.
